# Beneficial Effects of Qili Qiangxin Capsule on Lung Structural Remodeling in Ischemic Heart Failure via TGF-**β**1/Smad3 Pathway

**DOI:** 10.1155/2015/298631

**Published:** 2015-10-28

**Authors:** Yaoyao He, Bai Du, Huiting Fan, Jian Cao, Zi Wang Liu, Yonglie Zhao, Mingjing Zhao, Yizhou Zhao, Xin Zhao, Xiangning Cui

**Affiliations:** ^1^Department of Cardiology, Guang'anmen Hospital, China Academy of Chinese Medical Sciences, Beijing 100053, China; ^2^Beijing University of Chinese Medicine Third Affiliated Hospital, Beijing 100029, China; ^3^The Key Laboratory of Chinese Internal Medicine of the Ministry of Education, Dongzhimen Hospital, Beijing University of Chinese Medicine, Beijing 100700, China

## Abstract

Qili qiangxin (QL) capsule is a traditional Chinese medicine that is widely used for the treatment of patients with chronic heart failure (CHF) of all etiologies, although the exact mechanisms of action remain unclear. CHF leads to pulmonary vascular remodelling and thickening of the alveolar-capillary barrier that may be important mechanisms in the poor clinical outcome in patients with end-stage heart failure. We examined whether QL could improve lung injury in ischemic CHF by reducing lung remodeling. Rats with myocardial infarct received QL (1.0 g/kg/day) for 4 weeks. Echocardiographic and morphometric measurements were obtained followed by echocardiography, histological staining, and immunohistochemical analysis of lung sections. CHF caused significant lung structural remodeling evidenced by collagen deposition and thickening of the alveolar septa after myocardial infarct that were greatly improved by QL. Lung weight increased after infarct with no evidence of pulmonary edema and was normalized by QL. QL also reduced lung transforming growth factor-*β*1 (TGF-*β*1), p-Smad3, tumor necrosis factor-*α* (TNF-*α*), and Toll-like receptor-4 (TLR4) expression. Thus, QL reduces lung remodeling associated with CHF, mainly by suppressing the TGF-*β*1/Smad3 signaling pathway. The mechanism may also involve inhibition of TLR4 intracellular signaling.

## 1. Introduction

Pulmonary hypertension (PH) is a frequent complication of chronic heart failure (CHF). It is predicted that 60% of patients with severe left ventricular (LV) systolic dysfunction and 70% of patients with LV diastolic dysfunction may develop PH [1]. PH secondary to chronic LV failure reduces exercise capacity and represents an important independent prognostic factor in sufferers especially when associated with right ventricular (RV) dysfunction [2–4]. In view of the growing prevalence of CHF, the clinical importance of PH associated with CHF is becoming more prominent.

The pathophysiology of CHF-associated lung injury is incompletely understood, and few effective therapeutic options are presently available. Although fluid overload and pulmonary edema no doubt contribute to the process, the mechanisms responsible for the pulmonary manifestations of chronic CHF involve both pulmonary vascular and alveolar septa structural remodeling [5, 6]. Therefore, novel therapies specifically targeting the lung structural remodeling processes may potentially be utilized in the future.

Research has provided strong evidence implicating inflammatory activation as an important pathway in the progression of CHF. Elevated plasma levels of cytokines such as tumor necrosis factor-*α* (TNF-*α*), interleukin-1*β* (IL-1*β*), and interleukin-6 (IL-6) have been found in CHF patients [7, 8], and subsequent animal experiments have suggested that certain anti-inflammatory therapies may be beneficial. Various experimental studies have also shown that increased expression of inflammatory cytokines is associated with tissue and organ fibrosis remodeling, in the heart, lung, and kidney [9–11]. Anti-inflammatory therapies can prevent pathological fibrosis in these organs. Animal experiments have also demonstrated that lung injury associated with CHF is characterized by excessive collagen deposition in the alveoli, inside the vessels, and in the vascular walls of large vessels, accompanied by the accumulation of leukocytes and macrophages and increased levels of transforming growth factor-*β*1 (TGF-*β*1), TNF-*α*, and Toll-like receptor-4 (TLR4) mRNA or protein content in the lungs [12]. Thus, controlling excessive inflammation could potentially be of great therapeutic benefit for inhibiting the progressive pulmonary structural fibrosis remolding in CHF.

Qili qiangxin (QL) capsule is a traditional Chinese medicine that is widely used for the treatment of patients with CHF of all etiologies, including hypertension, coronary heart disease, and chronic obstructive pulmonary disease [13]. Our laboratory and others have reported that QL can reduce cardiac fibrosis remolding and improve cardiac function [14–16]. The use of QL in CHF also has an immunomodulatory effect by decreasing the proinflammatory cytokine TNF-*α* and increasing the anti-inflammatory cytokine IL-10 [17]. The use of QL in patients with CHF caused by chronic obstructive pulmonary disease has shown positive effects on pulmonary function by improving gas diffusion and exercise capacity [18], suggesting that QL could also have beneficial effects on lung fibrosis and inflammation in CHF. In the present study, we used a postmyocardial infarction heart failure model to determine whether QL can improve lung structural remodeling associated with CHF by inhibiting lung inflammation and fibrosis and to elucidate the possible mechanisms underlying the antifibrotic effect of QL. The effects of QL were also compared with Valsartan, an Ang-II receptor antagonist commonly used in clinical practice, which is known to have heart-lung protective effects in the treatment of CHF [6].

## 2. Materials and Methods

### 2.1. Vegetal Material

QL consists of ginseng, Radix Astragali, Aconite Root,* Salvia miltiorrhiza*, Semen Lepidii Apetali, Cortex Periplocae Sepii Radicis, Rhizoma Alismatis,* Carthamus tinctorius*,* Polygonati odorati rhizoma*, Seasoned Orange Peel, and Ramulus Cinnamomi (Yiling Pharmaceutical Corporation, Shijiazhuang, China). We dissolved the drug powder in sterile water at a concentration of 0.1 g/mL QL for the study. Valsartan (batch number X1428), manufactured by Beijing Novartis Pharmaceutical Co. Ltd., was dissolved in sterile water.

### 2.2. Animal Model and Administration

Healthy male Sprague-Dawley rats (body weight 220~250 g) were provided by Beijing Vital River Laboratory Animal Technology Co. Ltd. (animal license number: SCXK (Beijing) 2012-0001). The animals were fed a standard diet and water was provided ad libitum. The rats were maintained under a 12 h light and 12 h dark cycle, at a temperature of 20 ± 2°C, and at a humidity of 50 ± 2%. All animal experimental protocols were approved by the Animal Care and Use Committee of Beijing University of Chinese Medicine and complied with laboratory animal management and use regulations. The CHF model was induced by myocardial infarction following ligation of the left anterior descending artery (LAD). A 1% sodium pentobarbital solution (50 mg/kg) was administered by intraperitoneal injection. The procedures performed included endotracheal intubation, positive pressure ventilation using a ventilator, preoperative recording of a 12-lead ECG, local skin disinfection, chest opening, thoracotomy device setup, and opening of the pericardium, the pulmonary cone, and the left atrial appendage 2~3 mm from the bottom of the left anterior descending coronary artery ligation. For the rats assigned to the Sham group, the same operation was performed without ligation of the left coronary artery. A 12-lead ECG was recorded after the experiment. Rats with myocardial infarctions (MI) were fed normally for 4 weeks. Based on the transthoracic echocardiography results (Table 1), the surviving rats were randomly assigned to the following groups: Model group (CHF, *n* = 12), Sham group (Sham, *n* = 10), QL group (QL, *n* = 9), and Valsartan group (Valsartan, *n* = 9). QL (1.0 g/kg) and Valsartan (10 mg/kg) were administered by gavage once a day during the 4 weeks. An equal volume of distilled water was used for the Model and Sham groups. See Table 1: echocardiographic ejection fraction levels in the study groups before treatment with QL.

### 2.3. Transthoracic Echocardiography Measurements

A noninvasive transthoracic echocardiography method was used to evaluate the morphology and function of the left ventricle. Echocardiography was performed on anesthetized animals using a two-dimensional mode, that is, a time-motion (TM) mode, and blood flow was measured using the pulsed Doppler mode.

### 2.4. Sample Preparation

At the end of the 4-week treatment, the rats were anesthetized. The heart, lungs, and other major organs were harvested and weighed. The lung tissue was dried at 58°C to a constant weight. The relative water content of the lung tissue was calculated. The left lung and LV were snap-frozen in liquid nitrogen pending biochemical analysis. The airways of the upper right lobe and hearts were subsequently fixed in 4% paraformaldehyde and embedded in paraffin for histological analysis.

### 2.5. Histological Staining

The relative pulmonary vascular muscularization was determined using hematoxylin and eosin (H&E) stains. Briefly, in each mouse, 60 intra-acinar arteries were examined and categorized as nonmuscular (NM), partially muscular (PM), or fully muscular (FM). The relative percentages of NM, PM, and FM arteries were calculated. Lung and heart fibrosis areas were stained using Masson Trichrome Stain Kit from Sigma-Aldrich. In addition, lung sections were stained with monoclonal antibodies to identify smooth muscle cells or myofibroblasts (using a mouse monoclonal antialpha smooth muscle actin antibody, ABcam, Inc.). Paraffin was removed from 4 *μ*m sections of tissue using xylene, endogenous peroxidase was quenched in 3% H_2_O_2_ for 10 min, and then antigen was recovered in Tris-EDTA buffer (pH = 9.0) for 2 min, 30 s at 140°C then washed in phosphate buffered saline (PBS). The sections were incubated with monoclonal antialpha smooth muscle actin antibody (1 : 400) at 37°C for 1 h followed by biotinylated secondary antibody for 20 min. The sections were stained with DAB chromogen and counterstained with hematoxylin.

### 2.6. Western Blot Analysis

All animals were euthanized after 4 weeks of drug administration, and their hearts and lungs were immediately harvested and stored in liquid nitrogen until western blot analyses were performed. The following antibodies were used: mouse monoclonal anti-TGF beta 1 (1 : 500, ABcam, Inc.), rabbit monoclonal anti-Smad3 (phosphor C25A9) (1 : 500, Cell Signaling Technology, Inc.), rabbit polyclonal anti-TNF alpha (1 : 1000, ABcam, Inc.), and mouse monoclonal anti-TLR4 (1 : 2000, ABcam, Inc.). Proteins were separated using 10% SDS-PAGE and transferred to nitrocellulose membranes, which were then incubated with antibodies at 4°C. The membranes were further incubated with horseradish peroxidase-conjugated anti-rabbit IgG (1 : 2000) for 2 h at room temperature. ECL visualization was performed and the Gene Gnome Gel Imaging System (Syngene Co.) was used to capture the resulting images. Image J (NIH image, Bethesda, MD) was used to analyze the gel images.

### 2.7. Statistical Methods

All experimental data are presented as the mean ± the standard deviation (SD); single factor analysis of variance (ANOVA) was performed using SPSS version 17.0 statistical software, Dunnett's T3 was used for unequal variances, and a probability of *P* < 0.05 was considered statistically significant.

## 3. Results

### 3.1. Effects of QL on LV Function and LV Remodeling

**Figure 1:**
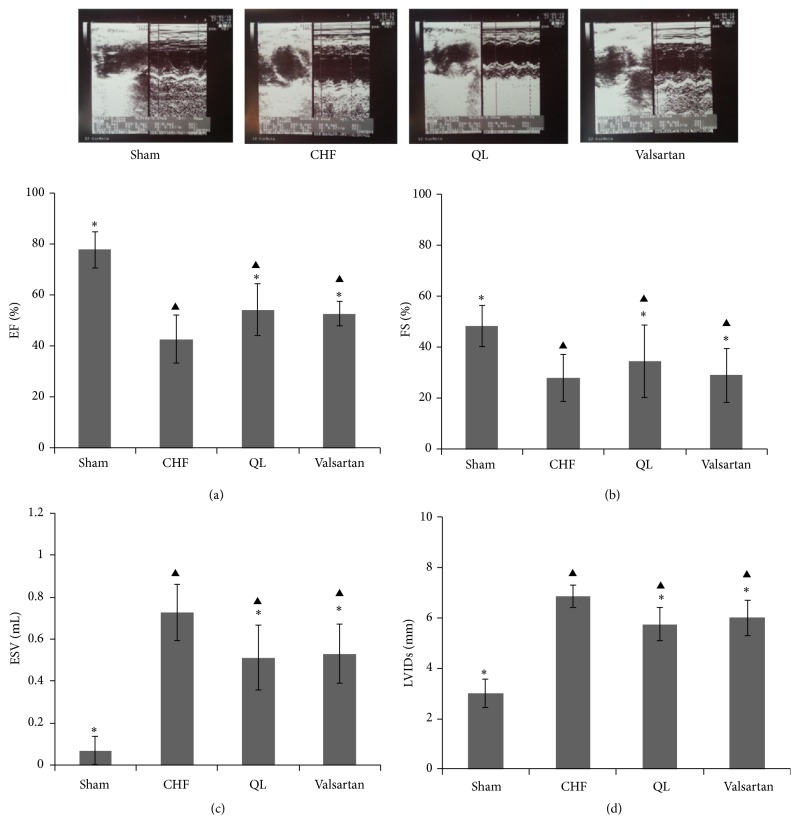
Effects of qili qiangxin (QL) on echocardiographic left ventricular (LV) function in rats with chronic heart failure (CHF). Typical echocardiography images (upper pictures) and (a) LV ejection fraction (EF), (b) LV fractional shortening (FS), (c) LV end-systolic volume (ESV), and (d) LV end-systolic dimensions (LVIDs) (^▲^ *P* < 0.05 versus the Sham group, ^*∗*^ *P* < 0.05 versus the CHF group).

**Figure 2:**
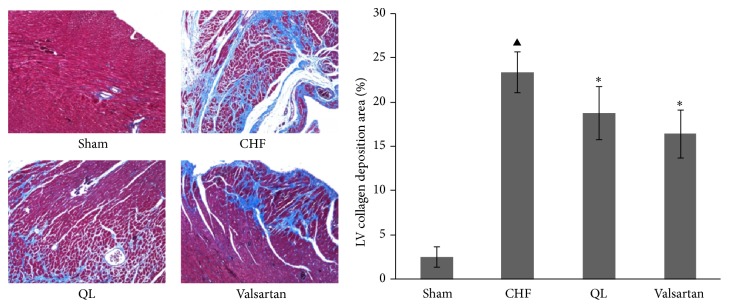
Effects of qili qiangxin (QL) on myocardial fibrosis in rats with chronic heart failure (CHF). Representative Masson Trichrome-stained left ventricular (LV) areas are shown. Blue areas indicate fibrotic staining. Fibrosis was measured in the whole LV section, and 5 sections for each heart were calculated (^▲^ *P* < 0.05 versus the Sham group, ^*∗*^ *P* < 0.05 versus the CHF group).

LV echocardiographic parameters are presented in Table 2. Compared with the CHF group (*n* = 11), the ejection fraction and fractional shortening measurements were elevated in the QL group (*n* = 8) and the Valsartan group (*n* = 8) (*P* < 0.05), while the end-systolic volume and LV end-systolic dimension measurements were lower (*P* < 0.05). Although the measurements obtained for end-diastolic volume (EDV) and LV end-diastolic dimension (LVIDd) displayed a decreasing trend in both the QL and the Valsartan groups versus the CHF group, the difference was not statistically significant (*P* > 0.05). Compared with those of the Sham group, the EF and FS measurements obtained from the CHF group, the QL group, and the Valsartan group were reduced (*P* < 0.05), while the ESV and LVIDs measurements were increased (*P* < 0.05) (Figure 1). Histological study revealed that the LV collagen fractional area (Figure 2) was highly increased in CHF versus Sham rats (*P* < 0.05) and was reduced by QL and Valsartan treatments. These parameters of LV remodeling and dysfunction were significantly modified by the QL treatment.

### 3.2. Effects of QL on Pulmonary Structural Remodeling

**Figure 3:**
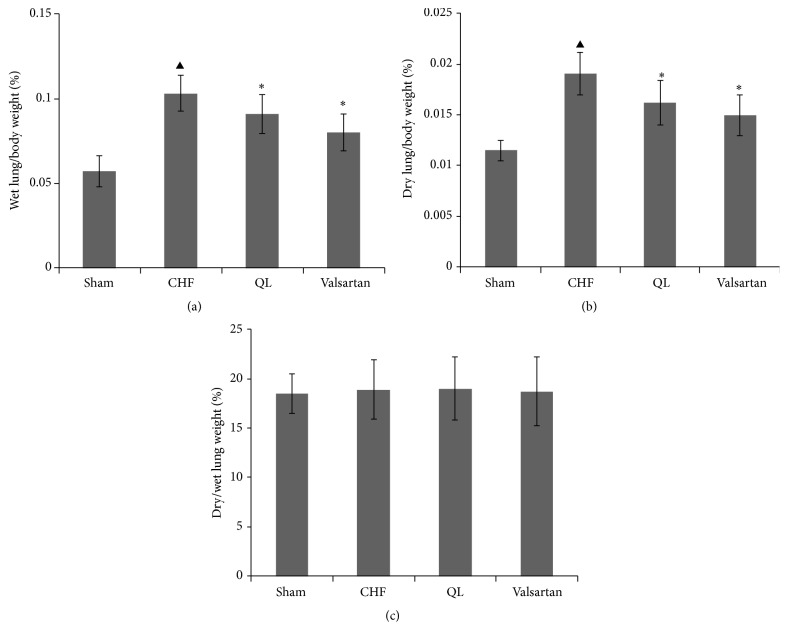
Effect of qili qiangxin (QL) on (a) wet lung/body weights, (b) dry lung/body weights, and (c) dry/wet lung weights ratios in rats with chronic heart failure (CHF) (^▲^ *P* < 0.05 versus the Sham group, ^*∗*^ *P* < 0.05 versus the CHF group).

The wet lung/body weight ratio increased after MI (*P* < 0.05) and QL and Valsartan markedly improved this ratio (*P* < 0.05, Figure 3(a)). Similarly, the dry lung/body weight ratio increased after MI (*P* < 0.05), providing evidence of substantial pulmonary remodeling; treatment with QL and Valsartan reversed this increased ratio (*P* < 0.05, Figure 3(b)). The dry/wet lung weight ratio was comparable among all groups, suggesting that no significant edema occurred (Figure 3(c)).

**Figure 4:**
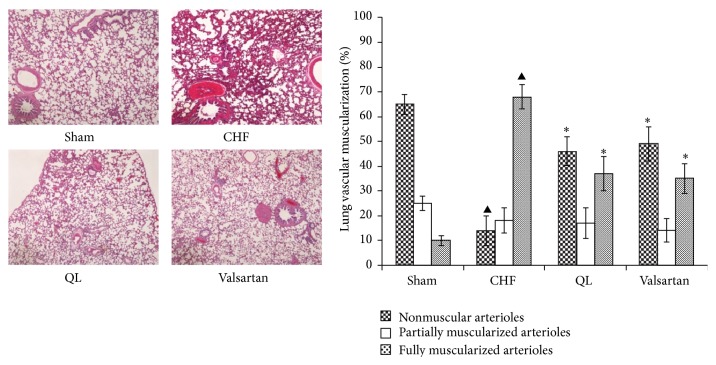
Effects of qili qiangxin (QL) on lung vascular muscularization in rats with chronic heart failure (CHF). Histological lung section with hematoxylin and eosin staining and quantitative analysis of the distribution of nonmuscular, partially muscular, fully muscularized small arteries (^▲^ *P* < 0.05 versus the Sham group, ^*∗*^ *P* < 0.05 versus the CHF group).

**Figure 5:**
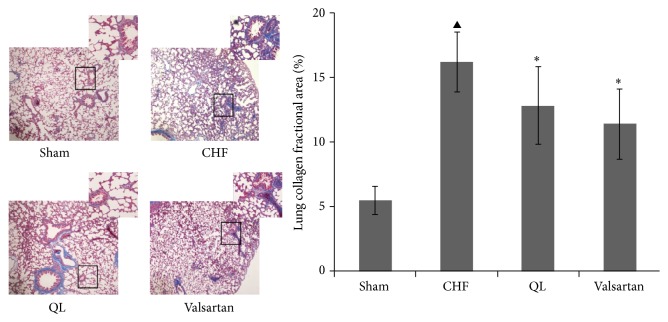
Effects of qili qiangxin (QL) on pulmonary structural remodeling assessed by Masson Trichrome staining for collagen in blue and by quantitative analysis for lung collagen deposition (^▲^ *P* < 0.05 versus the Sham group, ^*∗*^ *P* < 0.05 versus the chronic heart failure group).

**Figure 6:**
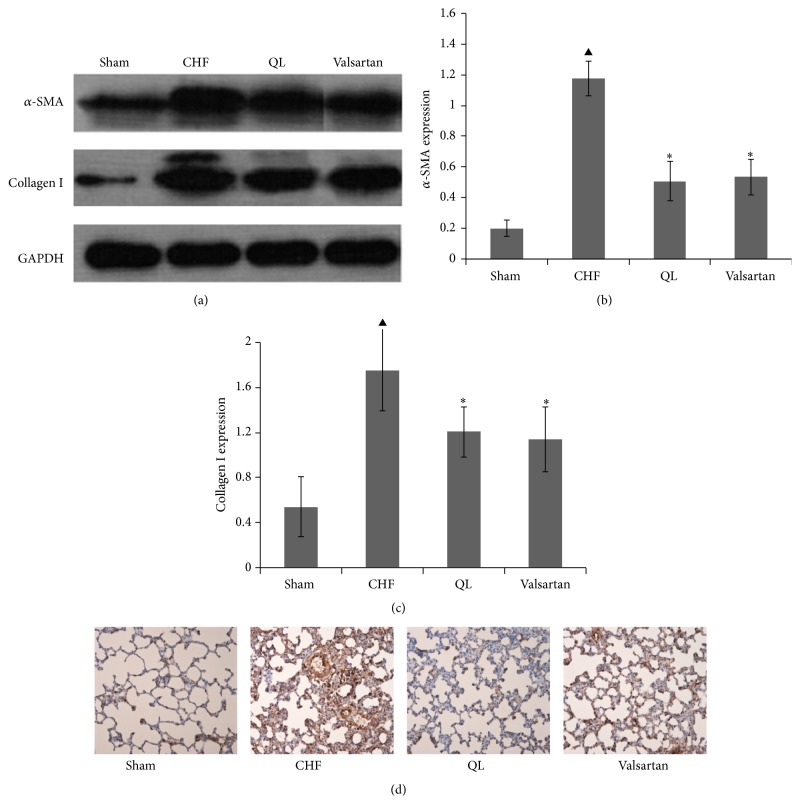
Effects of qili qiangxin (QL) on expression of proteins related to lung fibrosis in rats with chronic heart failure (CHF). ((a), (b)) Expression of smooth muscle *α*-actin (*α*-SMA). ((a), (c)) Expression of type I collagen. (d) Immunohistochemistry for *α*-SMA (^▲^ *P* < 0.05 versus the Sham group, ^*∗*^ *P* < 0.05 versus the CHF group).

The HE staining showed that intact and clear alveoli, normal interstitium, and few inflammatory cells were observed in the lungs of Sham group. However, LV dysfunction caused progressive lung injury, as demonstrated by destruction of lung alveoli, inflammatory cell infiltration, and thickening of the lung interstitium. QL or Valsartan treatment prevented these changes in rat lungs with MI. LV dysfunction also caused an increase in fully muscularized (FM) small arteries, but the number of FM small arteries was significantly lower among the rats in the QL and Valsartan treatment groups compared with those in the CHF group. Similarly, the QL treatment group exhibited a significant increase in nonmuscularized (NM) small arteries compared with the CHF group (Figure 4), indicating that QL and Valsartan significantly improved LAD-induced lung vascular remodeling. The Masson Trichrome staining also showed intact alveoli and normal interstitium in the lungs of the rats in the Sham group. LAD resulted in exacerbated lung alveoli destruction, interstitium thickening, and fibroblast diffusion in rat lungs, and QL or Valsartan treatment significantly halted these changes in rat lungs after MI (Figure 5). Alpha smooth muscle actin (*α*-SMA) expression has been extensively used as a marker of fibroblast differentiation into its activated state, the myofibroblast. Expression of *α*-SMA was increased in the interalveolar septa of animals with MI, when compared to the Sham group; however, QL or Valsartan treatment significantly reduced its expression (Figure 6).

The *α*-SMA-positive myofibroblasts actively synthesize ECM components. We then measured type I collagen, the main collagen isoform produced by fibroblasts in many fibrotic processes. Using western blot, we found that type I collagen deposition was significantly higher in the rats in the CHF group when compared to those in the Sham group; however, QL significantly decreased type I collagen protein expression in the lung (Figure 6). Considered together, these parameters of lung tissue fibrotic remodeling were significantly reversed by QL and Valsartan treatment.

### 3.3. QL Treatment Decreased TGF-*β*1/Smad3 Signaling Pathway Expression in Rat Lungs after CHF

**Figure 7:**
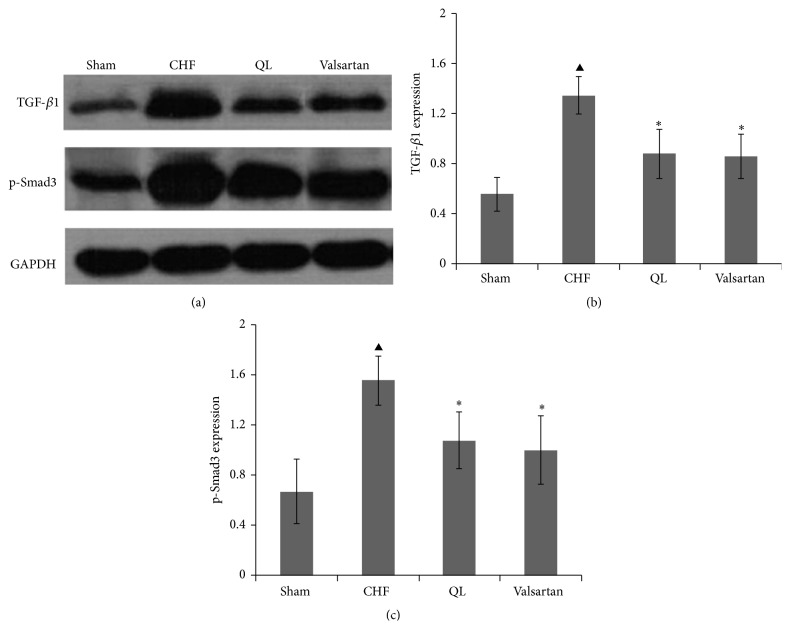
Effects of qili qiangxin (QL) on lung TGF-*β*1/Smad3 signaling pathway expression in CHF rats. ((a), (b)) Expression of transforming growth factor TGF-*β*1; ((a), (c)) expression of p-Smad3 (^▲^ *P* < 0.05 versus the Sham group, ^*∗*^ *P* < 0.05 versus the CHF group).

CHF evidently increased the protein levels of TGF-*β*1 in rat lungs compared with the Sham group at the endpoint of 28 days; however, QL and Valsartan treatments significantly reduced its expression compared with the CHF group (Figure 7). Similarly, CHF evidently increased the protein level of p-Smad3 in rat lungs compared with the Sham group, and QL or Valsartan treatment significantly reduced its expression compared with the CHF group (Figure 7).

### 3.4. QL Treatment Decreased TLR4 and TNF-*α* Expression in Rat Lungs after CHF

**Figure 8:**
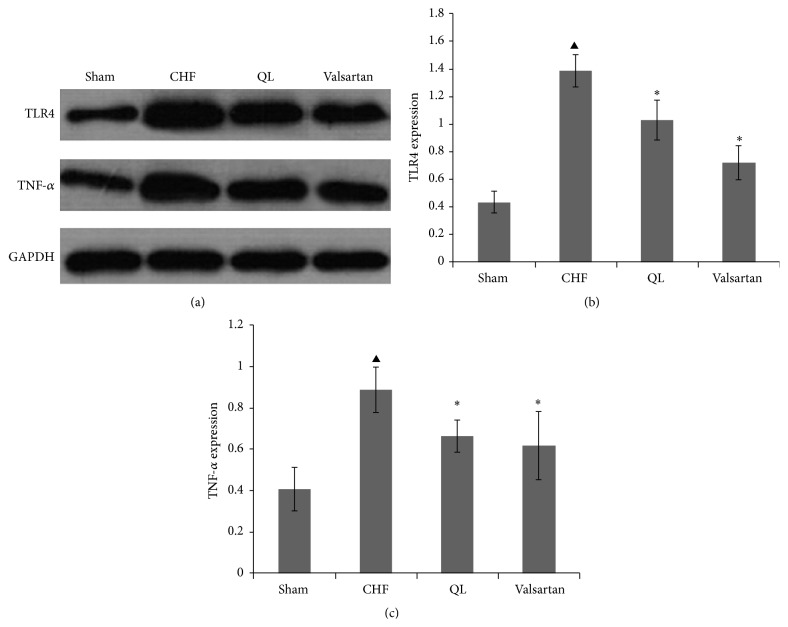
Effect of qili qiangxin (QL) on expression of protein related to lung inflammation in rats with chronic heart failure (CHF). ((a), (b)) Expression of Toll-like receptor-4 (TLR4). ((a), (c)) Tumor necrosis factor-*α* (TNF-*α*) (^▲^ *P* < 0.05, versus the Sham group, ^*∗*^ *P* < 0.05 versus the CHF group).

The protein expression of the proinflammatory cytokines tumor necrosis factor-*α* (TNF-*α*) and Toll-like receptor 4 (TLR4) was all increased in lung tissue from the rats in the CHF groups, and these increases were markedly reversed in the QL or the Valsartan treatment group rats compared with the CHF group rats (Figure 8).

## 4. Discussion

Adverse cardiac remodeling after MI leads to progressive heart failure. In the present study, we found that QL improves cardiac structure and function in rats with MI. After MI, rats developed CHF with important lung structural remodeling characterized by alveolar wall collagen deposition, a dramatic increase in the percentage of FM lung vessels, and increases in TGF-*β*1, TNF-*α*, and TLR4 proteins expression in lung tissues. Therapy with QL and Valsartan reduced lung tissue collagen deposition and the percentage of FM lung vessels, mechanistically; QL reduced lung TGF-*β*1, TNF-*α*, and TLR4 protein expression. These data suggest some direct beneficial effects of QL on pulmonary structure fibrosis, remolding, and inflammation response in rats with ischemic heart failure.

The lungs are the primary organs affected in CHF. CHF caused by all etiologies leads to impairment of LV filling with subsequent congestion of the pulmonary venous circulation and pulmonary alveolar-capillary stress failure, resulting in cycles of alveolar wall injury and repair. The reparative process causes the proliferation of myofibroblasts with fibrosis and extracellular matrix deposition, resulting in thickening of the pulmonary vascular and alveolar walls. Although the resultant reduction in vascular permeability is initially protective against pulmonary edema, the process becomes maladaptive causing a restrictive lung syndrome with impaired gas exchange. This pathological process may also contribute to type 2 PH (PH secondary to chronic LV failure is classified as type 2 PH). The structural fibrosis remodeling of the lung may be an important mechanism to explain the poor clinical outcome in patients with end-stage heart failure. Thus, the effective treatment of LV end-stage heart failure may also require additional efforts to reduce lung fibrosis and the inflammatory response.

QL capsule has been proven to be effective and safe for the treatment of CHF after MI [13, 19]. The compounds include 11 Chinese herbs in which Radix Astragali and ginseng comprise the main active constituents which have effects on invigorating the heart qi and lung qi.* Salvia miltiorrhiza* and* Carthamus tinctorius* accelerate blood circulation and relieve congestion. Semen Lepidii Apetali can relieve asthma and disperse lung edema. The use of these herbs demonstrated that QL has protective effects on the heart and lungs. Pharmacological studies have found that QL contains a number of active substances such as ginseng saponin, astragalus saponin, flavonoids, cardenolide, and phenolic acid which have been proven to have positive inotropic, vasodilation, anti-inflammation, and antifibrosis effects. In this study, we found excessive collagen deposition and upregulated *α*-SMA protein expression in the lungs of rats with CHF; both were reversed by QL and Valsartan therapy, suggesting that QL may also have pulmonary protective effects on ischemic heart failure.

To elucidate the possible mechanisms underlying the antifibrotic effect of QL in CHF, we examined the impact of QL on the TGF-*β*1/Smad3 signaling pathway. TGF-*β*1 is a locally generated cytokine that has been implicated as a major contributor to myofibroblast proliferation and tissue fibrosis in various organ systems. Several studies have explored the functional consequence of TGF-*β* in heart failure (HF), and results from these studies have shown that TGF-*β* antagonism inhibits fibrotic processes and provides salutary cardiac effects in HF [20]. As a main downstream signal transducer of TGF-*β*1, Smad3 can be phosphorylated by an activated type I receptor of TGF-*β*1, followed by forming a complex with Smad4 and translocation into the nucleus, where it acts as a transcription factor and promotes the expression of target genes including type I and type III collagen. The present study demonstrated that TGF-*β*1 expression in lung tissues of rats with CHF was increased, and the level of p-Smad3 was also markedly increased; both were reversed by QL therapy. These results suggest that the significant activation of TGF-*β*1/Smad3 signaling pathway in lung tissues after CHF can lead to myofibroblast proliferation and a marked upregulation of type I collagen expression, suggesting that QL could suppress the TGF-*β*1/Smad3 signaling pathway, which might contribute to its attenuation of lung fibrosis in CHF.

We also found that QL can reverse the increased TLR4 and TNF-*α* expression in lung tissues of CHF. TLR4, a member of the family of Toll-like receptors (TLRs), an important receptor of pattern recognition, can induce the secretion of many inflammatory cytokines including TNF-*α*. Endogenous TLR4 ligands not only are important in the regulation of innate and adaptive immune responses, but also are involved in noninfectious inflammatory and fibrotic diseases of the heart, lung, and kidney. Moreover, mice with genetic ablation of TLR4 are protected from experimentally induced inflammation and fibrogenesis in these organs, suggesting an important functional role of TLR4 in sustaining pathological fibrogenesis [20–24]. Although multiple mechanisms underlie these profibrotic effects of TLR4 in fibroblasts, enhanced Smad signaling, which normally limits the intensity and duration of fibrotic responses, appears to be prominent [25]. TNF-*α* is a proinflammatory cytokine that plays an important role in the activation of host defense and is associated with increased mortality in CHF patients and the development of pulmonary fibrosis [26, 27]. Sullivan et al. confirmed that TNF-*α* controls the expression of TGF-*β*1 synthesis throughout the MEK/ERK pathway and AP-1 activation [28, 29]. The present study demonstrated that TNF-*α* expression in lung tissues of CHF increased and was reversed by QL therapy. Thus, our results demonstrated that QL may exert its proinflammatory and profibrotic effect by modulation of TLR4 and downstream of TNF-*α* and TGF-*β*1/Smad3 signaling pathway.

## 5. Conclusions

The present study provides evidence that using QL for 4 weeks improves pulmonary structure remodeling in rats with post-MI congestive heart failure. Based on our results, we conclude that QL accomplishes its antifibrotic effects mainly by suppressing the TGF-*β*1/Smad3 signaling pathway, which might contribute to its attenuation of myofibroblast proliferation and collagen deposition. The possible mechanisms may involve inhibition of TLR4 intracellular signaling. Further studies should explore the effects of QL on myofibroblast proliferation and TLR4 intracellular signaling pathway in vitro to ultimately offer new avenues for the prevention and treatment of this and other related diseases.

## Figures and Tables

**Table 1:** Echocardiographic ejection fraction level in the study groups before treatment with qili qiangxin (QL) ($\overset{-}{x}\pm s$).

Group	*n*	EF (%)
Sham	10	84.490 ± 7.3354
CHF	12	44.708 ± 8.4369^*∗*^
QL	9	47.933 ± 9.1211^*∗*^
Valsartan	9	47.911 ± 9.1068^*∗*^

^*∗*^ *P* > 0.05 versus the Sham group.

**Table 2:** Echocardiographic left ventricular (LV) parameters in the study groups after treatment with qili qiangxin (QL) ($\overset{-}{x}\pm s$).

Group	*n*	EF (%)	FS (%)	LVIDd (mm)	LVIDs (mm)	EDV (mL)	ESV (mL)
Sham	10	77.8 ± 7.1	48.3 ± 8.2	5.9 ± 0.7	3.0 ± 0.6	0.49 ± 0.14	0.07 ± 0.07
CHF	11	38.7 ± 5.8^▲^	27.9 ± 9.3^▲^	7.4 ± 1.2^▲^	6.9 ± 0.4^▲^	0.92 ± 0.52^▲^	0.73 ± 0.13^▲^
QL	8	54.2 ± 10.3^*∗*^	34.3 ± 14.2^*∗*^	6.5 ± 1.2	5.8 ± 0.7^*∗*^	0.73 ± 0.23	0.51 ± 0.12^*∗*^
Valsartan	8	52.7 ± 4.8^*∗*^	28.9 ± 10.7^*∗*^	6.6 ± 0.9	6.0 ± 0.7^*∗*^	0.71 ± 0.37	0.53 ± 0.14^*∗*^

^▲^ *P* < 0.05 versus the Sham group, ^*∗*^ *P* < 0.05 versus the CHF group.
